# Bringing it home: expanding the local reach of dissemination and implementation training via a university-based workshop

**DOI:** 10.1186/s13012-015-0281-6

**Published:** 2015-07-04

**Authors:** Elaine H. Morrato, Borsika Rabin, Jeff Proctor, Lisa C. Cicutto, Catherine T. Battaglia, Anne Lambert-Kerzner, Bonnie Leeman-Castillo, Michelle Prahl-Wretling, Bridget Nuechterlein, Russell E. Glasgow, Allison Kempe

**Affiliations:** Colorado School of Public Health, University of Colorado Anschutz Medical Campus, 13001 E. 17th Place, Mail Stop B119, Aurora, CO 80045 USA; Colorado Research in Implementation Science Program, Adult and Child Center for Health Outcomes Research and Delivery Science, School of Medicine, University of Colorado Anschutz Medical Campus, Aurora, CO USA; VA Eastern Colorado Health Care System, Denver, CO USA; Denver Seattle Center for Veteran-centric Value-based Research (DiSCoVVR), Denver, CO USA; The Evaluation Center, School of Education and Human Development, University of Colorado Denver, Denver, CO USA; Clinical Science Graduate Program, University of Colorado Anschutz Medical Campus, Aurora, CO USA; National Jewish Health, Denver, CO USA; Institute for Health Research, Kaiser Permanente, Denver, CO USA

**Keywords:** Dissemination and implementation science, Training, Online education

## Abstract

**Background:**

Currently, national training programs do not have the capacity to meet the growing demand for dissemination and implementation (D&I) workforce education and development. The Colorado Research in Implementation Science Program (CRISP) developed and delivered an introductory D&I workshop adapted from national programs to extend training reach and foster a local learning community for D&I.

**Methods:**

To gauge interest and assess learning needs, a pre-registration survey was administered. Based on feedback, a 1.5-day workshop was designed. Day 1 introduced D&I frameworks, strategies, and evaluation principles. Local and national D&I experts provided ignite-style talks on key lessons followed by panel discussion. Breakout sessions discussed community engagement and applying for D&I grants. A workbook was developed to enhance the training and provided exercises for application to an individual’s projects. Day 2 offered expert-led mentoring sessions with selected participants who desired advanced instruction. Two follow-up surveys (immediate post-workshop, 6 months) assessed knowledge gained from participation and utilization of workshop content.

**Results:**

Ninety-three workshop registrants completed an assessment survey to inform workshop objectives and curriculum design; 43 % were new and 54 % reported a basic understanding of the D&I field. Pre-registrants intended to use the training to “apply for a D&I grant” (73 %); “incorporate D&I into existing projects” (76 %), and for quality improvement (51 %). Sixty-eight individuals attended Day 1; 11 also attended Day 2 mentoring sessions. In the 1-week post-workshop survey (*n* = 34), 100 % strongly agreed they were satisfied with the training; 97 % strongly agreed the workshop workbook was a valuable resource. All Day 2 participants strongly agreed that working closely with faculty and experts increased their overall confidence. In the 6-month follow-up evaluation (*n* = 23), evidence of new D&I-related manuscripts and grant proposals was found. Training materials were published online (www.ucdenver.edu/implementation/workshops) and disseminated via the National Institutes of Health (NIH) Clinical and Translational Science Awards Consortium. To sustain reach, CRISP adapted the materials into an interactive e-book (www.CRISPebooks.org) and launched a new graduate course.

**Conclusions:**

Local D&I training workshops can extend the reach of national training programs.

## Background

The aim of dissemination and implementation (D&I) in health research is to improve health outcomes by translating evidence-based research findings into clinical and public health practice. However, the oft-quoted statistic—“it takes an average of 17 years for new knowledge generated by randomized controlled trials to be incorporated into practice, and even then application is highly uneven” [1]—illustrates the challenge in achieving this aim. The Institute of Medicine wrote a decade ago in *Priority Areas for National Action: Transforming Health Care Quality* that:

“. . . the stark reality [is] that we invest billions in research to find appropriate treatments, we spend more than $1 trillion on health care annually, we have extraordinary knowledge and capacity to deliver the best care in the world, but we repeatedly fail to translate that knowledge and capacity into clinical practice” [2].

Compounding the problem has been historically limited the funding supporting implementation science; for every dollar spent in discovery, mere pennies have been spent learning how effective interventions can be better disseminated [3].

The good news is that D&I research is an emerging national priority [4]. The Department of Veterans Health Administration (VHA) has been an early leader in the field establishing the Quality Enhancement Research Initiative (QUERI) and publishing an implementation guide [5]. The Agency for Healthcare Research and Quality (AHRQ) has also funded research networks to accelerate the diffusion of health care research into practice and to find solutions to the problems of underuse, overuse, and disparities in implementation of preventive services [6, 7]. The National Institutes of Health (NIH) maintains an active portfolio of D&I research [8, 9] and with the NIH Common Fund supporting a new Healthcare Systems Research Collaboratory to conduct implementation-ready pragmatic clinical trials. This latter program is being conducted in partnership with health care systems to strengthen the relevance of the research results and to expedite translation into health practice [10]. Lastly, the Patient-Centered Outcomes Research Institute (PCORI), an independent, nonprofit organization created under the Affordable Care Act, has the strategic goal to speed the implementation and use of patient-centered outcomes research evidence. Its emphasis on D&I is reflected in its merit review criteria for project proposals [11] and recent publication of a dissemination and implementation framework [12]. PCORI is mandated to invest 20 % of its funding into dissemination and research capacity building [13], approximately $400 million annually, which is also stimulating the area of D&I research.

A key challenge for this emerging field is that training programs have not kept up with the capacity needed to meet the growing demand for D&I workforce education and development. D&I research in health comprises paradigms from diverse disciplines, including psychology, public health, communication, and marketing. Therefore, because it is not situated within one specific discipline, there are few university-based D&I training programs [14, 15]. Existing D&I learning opportunities and resources include the following: formal training programs for early-stage investigators [16, 17], university-specific graduate programs [18], an annual dissemination and implementation science conference now hosted by NIH and Academy Health [19], and publication of a definitive textbook for the field [20].

Of particular note, the NIH and VHA established the Training Institute for Dissemination and Implementation Research in Health (TIDIRH) in 2011 [15]. In its first year, 30 trainees were selected from a pool of 266 applicants indicating a greater demand for training opportunities than TIDIRH could meet. To enhance program sustainability and reach, the programs follow a “train the trainer” model and aimed to generate a network of faculty who would share TIDIRH learning at their home institutions in the form of educational events, mentoring, and collaborations. Other national and international training programs were established by colleagues in the School of Social Work at Washington University in St. Louis focusing on research in D&I in the context of mental health [17] and cancer [21] and one focusing on knowledge translation in Canada [22]. In addition to training institutes, other educational resources were also created such as regular cyber webinars [23, 24].

Despite these efforts, leaders from the National Institutes of Health Clinical and Translational Science Award (CTSA) programs found deficiencies in D&I knowledge and practice, particularly at the local institutional level [25]. For example, one-third of respondents to a needs assessment survey conducted by the Colorado Clinical and Translational Science Institute was involved in translation to practice and population; however, half reported they “needed but either did not know this resource existed or how to access it” for D&I research training and consultation [26].

Therefore to bring D&I training to more local investigators, the Colorado Research in Implementation Science and Prevention Program (CRISP) at the University of Colorado Anschutz Medical Campus developed and delivered an introductory D&I training workshop involving leaders from the national TIDIRH program. This paper describes the context of the local training environment, findings from a pre-workshop needs assessment survey, training design and structure, and post-workshop evaluation. Lessons learned may inform others intending to develop local D&I training workshops.

## Methods

### Local institutional context

CRISP is an Agency for Healthcare Research and Quality (AHRQ) Research Center for Excellence in Clinical Preventive Services. It is a program that brings together expertise from across the CU Anschutz Medical Campus in implementation science [27–31], development of interventions to improve use of clinical preventive services [32–34], pragmatic research involving practice-based research networks (PBRNs) [35, 36], and health information technology (HIT) [37, 38]. CRISP’s overall aim is to expand understanding of how to effectively implement interventions that work in order to improve the appropriate use of clinical preventive care for children and adults in the US. CRISP focuses its implementation efforts on strengthening partnerships between primary care providers, public health entities, and communities.

CRISP is a program within the Adult and Child Center for Health Outcomes Research and Delivery Science (ACCORDS). ACCORDS serves as a local incubator for research ideas and fosters interdisciplinary collaboration between clinicians, social scientists, and health services researchers. ACCORDS functions as both an actual and virtual center, with a group of investigators from multiple disciplines who have their primary office on-site and a much larger group affiliating with ACCORDS personnel, programs, and cores while maintaining an off-site research home. Currently, 46 investigators, 25 research assistants, 12 biostatisticians/analysts, and 9 administrative and information technology personnel are co-located together at the ACCORDS center. Over a hundred additional investigators interface with the program, primarily for consultation or to attend educational offerings. Collaborating investigators represent all School of Medicine departments, as well the School of Public Health, the School of Pharmacy, and the College of Nursing. Clinical affiliations include the Colorado Children’s Hospital, University of Colorado Health System, Kaiser Permanente, Denver Health Authority, and the Veterans Health Administration.

One of AHRQ’s charges to its Research Centers for Excellence was to train researchers in implementation science [39]. CRISP organized its educational efforts into four domains by adapting knowledge frameworks used in implementation science [18, 20]: (1) theory and strategies, (2) community and stakeholder engagement, (3) implementation tools and approaches, and (4) evaluation, design, and analysis. A new seminar series on implementation science were initiated to foster a collaborative learning community. Based on key informant discussions with senior leadership on the Anschutz Medical Campus, it was determined that an introductory training workshop on implementation science would be a valuable next step in kick-starting knowledge quickly for a broad group of interested investigators, students, and research staff.

### Learning needs

To gauge workshop interest and assess D&I skill level and learning priorities, an online “save-the-date” registration survey was emailed 6 months ahead of the planned workshop to local university and health care organizations affiliated with CRISP and the Colorado Clinical and Translational Sciences Institute (CCTSI). The CCTSI is a collaborative enterprise between the Anschutz Medical Campus, University of Colorado Boulder, Colorado State University, six affiliated hospitals and health care organizations, and multiple community organizations.

**Fig. 1:**
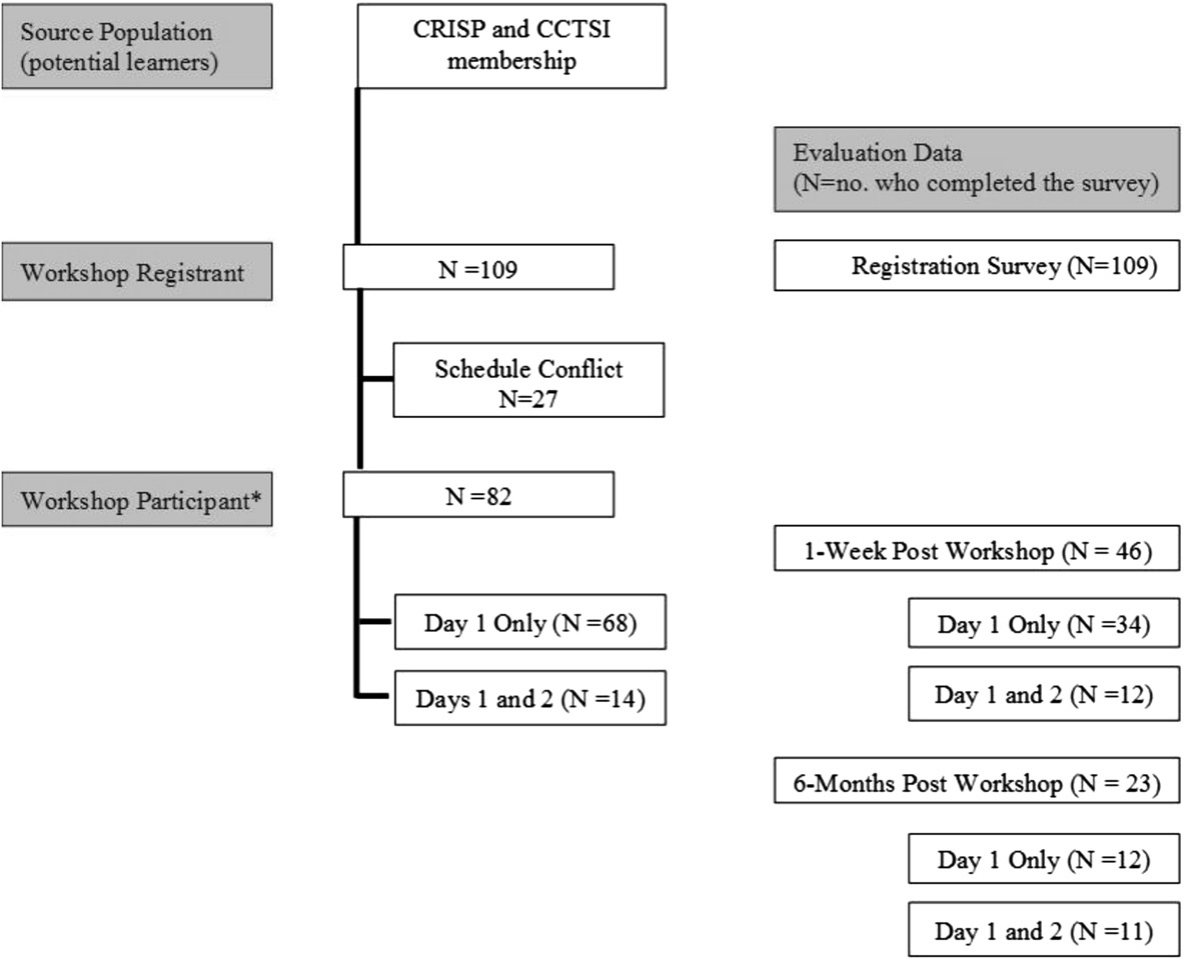
Flow diagram of D&I workshop registrants, participants, and evaluation data. *All workshop participants attended the full-day session on Day 1. Some participants self-selected to also attend the half-day session involving small-group consultation with experts on Day 2. CRISP indicates Colorado Research in Implementation Science Program; CCTSI, Colorado Clinical and Translational Sciences Institute

Figure 1 shows a flow diagram of participants who pre-registered and participated in the training workshop. The registration survey (Table 1) collected information on current D&I knowledge, intended application of workshop learning, preferred learning topics, and demographic characteristics. Forty-six percent of workshop registrants reported they were new to D&I science; 58 % reported a basic understanding of the concepts but wanted more training to increase their competitiveness in grant applications (note: respondents were able to select more than one choice for each question). The majority of registrants were primary investigators (62 %) with doctoral-level training (38 % PhD/DrPH, 36 % MD) affiliated with the School of Medicine (52 %). Of these respondents, only 3 % reported advanced training in D&I theory and methods.

**Table 1:** Registration survey results

Selected characteristics of workshop registrants	*n*	%
Educational Degree		
MD	39	36
PhD/DrPH	41	38
MPH/MSPH, MS	38	35
Other	25	23
Organizational affiliation		
Anschutz Medical Campus	52	57
School of Medicine		
Colorado School of Public Health	17	18
Other schools and colleges (e.g., pharmacy, nursing, dental medicine)	12	13
Healthcare facility		
Kaiser Permanente Colorado, National Jewish Health, Denver Health	14	16
Veterans Affairs	20	18
Denver Health	13	12
Children’s Hospital of Colorado	13	12
Other		
Type of affiliation		
Faculty	67	73
University research staff	7	8
Graduate student/fellow	14	15
Practicing clinician, health provider, or public health professional	14	15
Other	12	13
Highest role in research		
Principle investigator	62	66
Co-investigator/consultant	20	21
Other role	14	15
Led quality improvement projects	3	3
None	2	2
Level of D&I science knowledge		
New to D&I science	43	46
Basic understanding	54	58
Advanced training	3	3
Intended application of workshop learnings		
Apply for a D&I-related grant	73	77
Incorporate D&I principles into existing projects	76	80
Quality improvement practice	51	53
Other	9	9

*n* = 109. Percentages may add up to more to 100 % due to rounding, and participants were able to select more than one choice for each question

Most registrants intended to use the knowledge gained from the workshop to apply for grants, to develop D&I interventions, and to incorporate D&I practices into existing research projects. The top five topics of greatest interest were the following: D&I study design, D&I strategies, measurement for D&I, available tools and resources for D&I, and the theoretical basis for D&I science and practice. The registration survey also highlighted the training needs for two other local audiences: [1] clinical and public health practitioners who wanted to incorporate D&I principles into quality improvement initiatives and [2] research staff, who were not principal investigators, but wanted to better understand the field and expand their competencies. Half of the pre-registered participants wanted a 1-day workshop format.

### Learning objectives

Information from the registration survey was used to identify the learning objectives and delivery method for the workshop. As a result, the workshop was designed to be relevant to both researchers and practitioners interested in D&I for the improvement of public health and health care. The primary learning objective was to introduce participants to D&I concepts, strategies, and design principles. The goal was to provide the resources necessary for participants to formulate their own D&I research questions and to incorporate D&I principles into their grant proposals. The secondary learning objective was to build a stronger network of local D&I researchers and encourage cross-disciplinary mentorship and collaborations across the institution.

### Curriculum

Based on the audience who registered for the workshop, the curriculum was designed to meet the educational needs of both new learners and intermediary learners with basic knowledge of D&I principles. The workshop used the D&I definitions employed by NIH [8]:

- Dissemination: the targeted distribution of information and intervention materials to a specific public health or clinical practice audience. The intent is to spread (“scale up”) and sustain knowledge and the associated evidence-based interventions.
- Implementation: the use of strategies to adopt and integrate evidence-based health interventions and change practice patterns within specific settings.
- Dissemination and implementation research: research to produce a generalizable knowledge base about how health information, interventions, and new clinical practices and policies are transmitted and translated for public health and health care service use in specific settings.

Educators have emphasized that competency-based education in research and practice-oriented training programs is important [40, 41]. Padek and colleagues reported on developing differentiated D&I competencies for beginner, intermediary, and advanced learners at the 7^th^ Annual Dissemination and Implementation Conference sponsored by NIH and others [41]. This introductory workshop covered the following D&I competencies by domain:

- Definitions, backgrounds, and rationale. Describe the importance of D&I research and practice in achieving a healthy America. Define common D&I terminology.
- Educational content for this domain was obtained from the following sources: the National Institutes of Health on their priorities and approaches to D&I science [3], the QUERI organizational framework for system-level change [42] and implementation guide [5], PCORI’s national priorities and research agenda [43], and the chapter on D&I terminology by Rabin and Brownson in Dissemination and Implementation Research in Health and content from the www.makeresearchmatter.org website.
- Theories and approaches. Demonstrate the use of common D&I frameworks. Demonstrate how to design for D&I. Identify existing D&I resources and toolkits.
- Information on D&I models and frameworks was obtained from work published by investigators from the CDC Prevention Research Center at Washington University in St. Louis [44, 45]. Key models from diffusion theory [14, 46, 47] and referenced by the Implementation Science Division of the National Cancer Institute [48–53] were presented. Methods and approaches in stakeholder engagement were adapted from work supported through the NIH CTSA program [35, 54, 55]. Resources on D&I planning tools included the following: www.makeresearchmatter.org, the AHRQ dissemination planning tool in patient safety [56], and the Academy Health decision guide for researchers navigating the translation and dissemination of findings [57].
- Design and analysis. Compare and contrast study evaluation approaches commonly used in D&I. Identify key metrics in D&I.
- Resources on pragmatic trials and the PRECIS indicator to dimension more pragmatic trials [58–61] were used to describe common study design approaches. Resources on pragmatic measures [62], a conceptual framework for outcomes for implementation research [63], and cost measures to enhance translation [64] were discussed in the workshop.
- Practice-based considerations. Share tips for success in writing a D&I research proposal and incorporation of stakeholder engagement.
- Educational content from this domain was derived from the personal experiences of the expert faculty involved in the workshop (see Table 3).

The workshop was a condensed learning format so the planning committee also developed a companion workbook and online website for the participants as a take-home resource. The purpose of the workbook was not to replicate text from the workshop presentations but instead to serve as a compilation and navigator of D&I resources. It provided executive summaries of the material taught, tips for success and consolidated links to further resources. At the end of each section was a checklist for action, and some sections also provided self-learning activities. Printed workbooks were available at the workshop, and a free interactive online e-book was made available along with a link to an open-access online learning system (Canvas) on which all workshop presentations, documents, and videos were posted.

- Course materials available at: https://canvas.instructure.com/courses/810394
- Workbook available at: www.CRISPebooks.org

### Workshop structure and educational delivery

Based on the information from the needs assessment survey, a 1½-day introductory training workshop was developed. Day 1 provided an introduction to D&I principles for both beginner and intermediate learners. Day 2 was an optional half-day for learners who were more advanced and desired consultation with faculty and experts on a specific D&I project or proposal.

#### Day 1 (full day): an introduction to D&I science

Table 2 shows the agenda for Day 1. The overall philosophy of Day 1 was to bring the science to practical application. Many of the topics were covered in a mini-lecture format (e.g., 15 min). To facilitate discussion and cover a breadth of research topics, panel and moderation formats were also utilized. Additionally, there was an afternoon breakout session with three groups divided according to interest area: research, practice, and project management. To allow time for networking, participants had lunch in small groups and a reception for all participants followed the workshop.

**Table 2:** Workshop agenda for Day 1: an introduction to D&I Science

Topic areas	Learning objective	Time
Introduction	To review workshop objectives	8:00–8:10
Why are we here?	To describe the importance of D&I research and practice in achieving a healthy America	8:10–8:30
The promise and challenge of D&I in health		
What are we talking about?	To define common terminology	8:30–9:45
D&I definitions, frameworks	To demonstrate the use of common frameworks	
Break	To network with D&I colleagues	9:45–10:00
What approaches should I take?	To identify existing D&I resources and toolkits	10:00–11:30
Strategies and toolkits	To demonstrate how to design for D&I	
Lunch	To network with D&I colleagues	11:30–12:30
How do I know if I am successful?	To compare and contrast study evaluation approaches commonly used in D&I	12:30–2:00
Evaluation and measurement	To identify key metrics in D&I	
Break	To network with D&I colleagues	2:00–2:15
Application: RE-AIM activity	To apply knowledge of RE-AIM framework	2:15–3:15
Tips for success	To share tips for success in writing a research proposal, implementing a D&I program, or managing a D&I project	3:15–4:15
Summary	To recap key learning points	4:15–4:30
Reception	To network with D&I colleagues	4:30–5:30

#### Day 2 (half day): small-group consultation with D&I experts

The objective of Day 2 was to meet the needs of more advanced learners and provide opportunity for in-depth feedback on specific D&I proposals or projects. Participants were asked to submit a one- to two-page concept paper for one of the three D&I topic areas: (1) research, (2) practice, or (3) project management. This approach was modeled after the application process for NIH training institutes. Day 2 was a half-day session and participation was limited to allow for more in-depth small-group discussions with workshop faculty and invited experts. Groups were formed based on related research interests and study populations to facilitate peer-to-peer learning and mentorship. At the end of the session, the group facilitators met collectively to review common themes and design issues across projects.

### Workshop faculty

A workshop planning committee (comprised of the authors) was established to develop educational objectives and course content. Committee members represented a diverse cross section of local academic and health care system researchers in implementation science from the University of Colorado Schools of Medicine and Public Health; the Veterans Health Administration, Kaiser Permanente Colorado, and National Jewish Health.

A critical component of the workshop was involvement of local and national D&I experts to provide ignite-style short presentations and serve on expert discussion panels (Table 3). This served two learning objectives: to make the concepts more concrete for new learners using real-world case examples and to provide opportunity for in-depth and more nuanced dialogue for more intermediate and advanced learners. In addition, several of the expert faculty are involved in directing national D&I training programs and in the scientific review of D&I grant proposals. In this regard, they also provided feedback to the planning committee on adapting the curriculum and training materials for local use.

**Table 3:** Expert faculty

Guest faculty: D&I experts	Title/affiliation
Juliana Barnard, MA	Senior Professional Research Associate, University of Colorado Denver School of Medicine
Arne Beck, PhD	Senior investigator and Director of Quality Improvement and Strategic Research, Kaiser Permanente Colorado Institute for Health Research
Ross Brownson, PhD	Professor and Co-Director, CDC Prevention Research Center in St. Louis, Washington University in St. Louis
Russell Glasgow, PhD	Deputy Director Emeritus of Implementation Science, Division of Cancer Control and Population Sciences at the U.S. National Cancer Institute
David Goff, MD PhD	Dean, Colorado School of Public Health
Allison Kempe, MD, MPH	Director, Adult and Child Clinical Outcomes Research and Service Delivery Science Program, University of Colorado Denver
Grant Jones	Executive Director, The Center for African American Health
Julie Lowery, PhD	Associate Director, VA Ann Arbor Health Services Research & Development Center of Innovation
Spero Manson, PhD	Director, Center for American Indian and Alaska Native Diabetes Translational Research, Colorado School of Public Health,
Wilson Pace, MD	Chief Executive Officer, Distributed Ambulatory Research in Therapeutics Network (DARTNet) Institute
Debra Ritzwoller, PhD	Senior Investigator and health economist, Kaiser Permanente Colorado Institute for Health Research

### Evaluation and discussion

Figure 1 shows the number of workshop participants who provided feedback in the 1-week and 6-month follow-up evaluation surveys. The intent of these surveys was to provide formative feedback and guide changes for subsequent workshops. The CCTSI Evaluation Core conducted an independent evaluation to ascertain participant feedback and workshop outcomes. The survey questions elicited feedback about the workshop overall, knowledge gained, intended application of learnings, and suggestions for future workshops using closed and open-ended questions.

### 1-week post-workshop evaluation

**Fig. 2:**
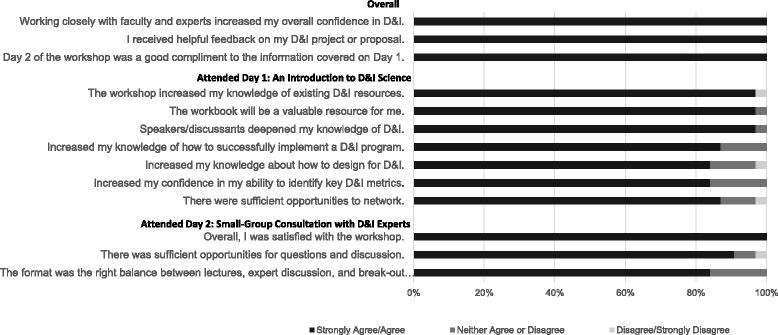
One-week post-workshop evaluation survey results

This evaluation assessed knowledge gained and feedback on workshop delivery. Survey response rates among those who attended Day 1 only was 50 % (*n* = 34 out of 68 participants) and 86 % (*n* = 12 out of 14 participants) for those who also attended Day 2. Figure 2 presents evaluation results. Overall, the feedback was positive. More than 90 % of the respondents agreed or strongly agreed that they were satisfied with the workshop; 100 % agreed or strongly agreed that this workshop increased their overall knowledge in D&I science. After attending this D&I workshop, 90 % of respondents reported that their knowledge increased “some” to “a lot” in the following areas: (a) D&I theory and definitions, (b) D&I programs/interventions, and (c) evaluating D&I programs/interventions. One individual expressed that this was a “great conference—very helpful, [and] well organized. [The] lectures were a nice mix of practical info[rmation] and theoretical/scientific info[rmation]. The workbook was a brilliant idea and a big bonus! [It] would be great if we could access more of these to distribute to researchers, fellows and other trainees.”

#### Day 1 feedback

Respondents most often commented on the value of presentations by experts in the field. One individual, new to the field, shared that he/she “found it to be a great overview of the material.” More specifically, “It was great to have leading experts presenting and giving insight.” Additionally, respondents provided a great deal of positive feedback regarding the usefulness of the materials and resources provided. Nearly all (97 %) of the respondents agreed or strongly agreed that the workshop workbook would be a valuable resource for them. One respondent stated that “the synthesis of literature and resources condensed into one source document [was] very helpful.”

#### Day 2 feedback

All respondents agreed or strongly agreed that Day 2 was beneficial. Specifically, 100 % of respondents agreed or strongly agreed that they received helpful feedback during the second day of the workshop. One individual stated that “the group discussion and feedback session was very helpful, both to learn from hearing about other D&I research and to get feedback on my own [research].” Also, respondents found it beneficial to discuss their projects with one another. For example, one individual stated he/she benefited from “meeting with others who are interested in similar types of research across multiple disciplines and hearing about problems and solutions others are currently facing.”

#### Suggestions for future D&I training workshops

When respondents were asked to recommend topics for future D&I workshops, the most common suggestion was to continue providing seminars/sessions similar to Day 2 during which participants could receive feedback on their projects or proposals. Topical suggestions included seminars on topics related to methodology, design and implementation, and measurement/assessment. One individual suggested,

“A dissemination type forum so that others can view applied D&I methods/findings and where you can also obtain feedback on your study.” Across items, various respondents suggested providing breakout sessions that are “geared more towards advanced users”

When designing D&I workshops in the future, one respondent suggested “having a ‘new to D&I day’ and an intermediate-advanced day, or have breakout sessions that cover the range of experience.”

### 6-month post-workshop evaluation

This formative evaluation assessed short-term outcomes from participating in the workshop. Survey response rates among those who attended Day 1 only was 18 % (*n* = 12 out of 68 participants) and 79 % (*n* = 12 out of 14 participants) for those who also attended Day 2. Note that the response rate was higher among those respondents who participated in the more in-depth, hands-on Day 2 session. This would be expected as they were more invested in the program.

Respondents reported that the most impactful/useful component of the workshop were “the new ideas/insights gained”. Approximately one-third of respondents reported that they had developed or were currently developing a proposal to obtain funding for a D&I relevant research project (*n* = 7). One-third reported they developed or were developing a D&I scientific paper (*n* = 7). Several mentioned (*n* = 4) they had modified a currently funded research project based on information from the workshop. One-third (*n* = 8) mentioned they had developed new D&I relevant collaborations and had attended multiple CRISP-sponsored seminars (*n* = 7). A majority of respondents (*n* = 10) had accessed D&I-related online resources and local institutional support since the workshop to expand upon their learning.

Future training suggestions included the following: (a) pragmatic clinical trial design, (b) advanced workshops on specific D&I topics, and (c) a refresher D&I workshop. Finally, survey respondents, who predominantly described themselves as having moderate D&I understanding, reported low to moderate interest in enrolling in a D&I research in health graduate course. This formative information led the CRISP education team to focus its next workshop on pragmatic trials because the majority of respondents (*n* = 12) reported being the most interested in this learning topic.

#### Addressing limitations

The purpose of the workshop was to engage both new and intermediate learners. An advantage of this approach was that it rapidly jump-started D&I training across the campus making it broadly visible. It provided efficiencies of scale for the workshop faculty allowing them to pool resources and attract D&I experts for a singular training event. Including learners at different levels also supported the idea of a learning community. A disadvantage of this approach is that it is challenging to provide differentiated learning in a condensed large-group setting. The use of expert panel discussion, luncheon breakout sessions, provision of a synthesizing workbook (then e-book) resource, and offering a second day for small-group discussion and expert feedback helped to address this challenge.

The evaluation is limited by a low response rate, particularly at the 6-month post-workshop time point, among participants who only participated in the first day of the workshop. It should be noted that response was high (79–86 %) for the group who also attended the more intensive second-day workshop. This may reflect their greater engagement in the learning objectives. On the positive side, evidence of new D&I grants and publications as a result of the workshop was found. However, the findings should be interpreted cautiously as their generalizability to all workshop participants is not known. Future evaluations should examine training outcomes by initial participant level of D&I knowledge (e.g., beginner vs. more advanced) and discipline or field.

Another limitation of an introductory workshop format is the inability to provide in-depth training and sustained research mentorship. To address this need, participants were directed to additional local resources, such as the monthly CRISP seminar series on D&I theory, methods, application and evaluation, and a new D&I graduate course offered through the Clinical Science program of the CCTSI (entitled Dissemination and Implementation Research in Health). The graduate course is targeted for beginning D&I learners and its educational objectives are similar to the workshop. It differs from the introductory workshop in that it allowed for more in-depth class discussion and reflection on the theory and measures and provided the forum for personal application of these concepts via a multi-step, graded assignment on the development of a dissemination and implementation plan. The course was first taught in the Fall 2015 semester to a group of students with diverse educational and background and research interests (e.g., clinical and public health). It received positive feedback and several faculty and students interested in D&I research are now writing this course into their education plans for their degree program, post-doctoral training, or career development grant proposals.

The introduction to D&I workshop catalyzed training activities at the Anschutz Medical Campus. Table 4 presents an overview of the CRISP educational resources that were introduced after the workshop by topic, mode of delivery, and intended audience. The seminar series regularly attracts approximately thirty attendees and helps to sustain a learning research community on campus and present more advanced methods and application. Based on feedback from the D&I workshop participants, two new training workshops were subsequently offered: design of pragmatic clinical trials and use of mHealth interventions (an advanced D&I topic). In addition, the CCTSI launched a Research Studio Program to provide targeted, structured, and collaborative discussion with multi-disciplinary experts addressing specific questions at a specific stage in the research process. Modeled after the program developed by Vanderbilt Institute for Clinical and Translational Research [65], it provides the opportunity for new and experienced D&I researchers to receive the tailored in-depth feedback they desire. The CCTSI Community Engagement Core also provides individual consultation and immersion training on best practices in conducting community-based participatory research.

**Table 4:** Implementation science educational resources at the University of Colorado Anschutz Medical Campus

		Audience				Year of offering			
Topic	Focus	B	I	A	Mode of delivery	2012	2013	2014	2015^a^
**CRISP resources**									
Seminar series	Methods and application.	X	X	X	In-person (1x/mo). Archived webinars.	X	X	X	X
	Fostering a learning community.								
*Implementation science*									
Workshop	Intro to theory, strategies, and evaluation. Case application. (Optional: expert consultation).	X	X	(X)	In-person (1 + 1/2 day). Open-access online learning system (Canvas).		X		
Resource e-book	Self-guided navigation of theory, strategies, and evaluation resources.	X	X	X	Interactive, web-based (www.CRISPebooks.org)			X	X
Graduate course^b^	Intro to theory, strategies, and evaluation. Proposal development.	X	X		In-person (2-credit hours). Graded assessment.			X	X
*Pragmatic trials*									
Workshop	Intro to PRECIS-2 [66] and patient engagement. Case application. (Optional: expert consultation).	X	X	(X)	In-person (1 + 1/2 day). Open-access online learning system (Canvas).			X	
Resource e-book	Self-guided navigation of PRECIS-2 and patient engagement resources.	X	X	X	Interactive, web-based (www.CRISPebooks.org).				X
*Special topics*									
mHealth workshop	Implementing and creating evidence for mHealth interventions. Developing mobile applications in the private sector.	X	X		In-person (1 day). Open-access online learning system (Canvas).			X	
PCORI research and engagement	Tips-for-success in PCORI research and engagement applications.	X	X		In-person (2 h). Open-access online learning system (Canvas).				X
**CCTSI resources**									
Community engagement	Facilitating community-based participatory research. Building capacity in community-academic partnerships.	X	X	X	Consultation. Five-day community immersion training and directed reading.	X	X	X	X
Research studio	A structured, collaborative roundtable discussion with relevant research experts [67]	X	X	X	Consultation (90-min).			X	X

CRISP indicates the Center for Research in Implementation Science and Prevention, a program of the Adult and Child Center for Health Outcomes Research and Delivery Science; CCTSI, Colorado Clinical and Translational Sciences Institute

*B* beginner, *I* intermediate, *A* advanced

^a^Planned

^b^Co-developed with the CCTSI

A new needs assessment survey is being fielded among the CRISP learning community to identify further training opportunities for differentiated D&I learning for beginner, intermediate, and advanced learners.

## Conclusion

A short D&I training workshop delivered in a local academic setting can extend the reach of national D&I training programs and quickly engage new and intermediary learners. Workshop success was strengthened by the use of a pre-workshop registration survey to guide curriculum development and the inclusion of national and local D&I experts. For the more experienced learners, the Day 2 intensive small-group feedback sessions were well received and a good complement to Day 1’s introductory nature. Convening the workshop also served as a catalyst for building a shared educational vision among diverse faculty across the organizations with D&I expertise at our local institution (i.e., schools of medicine, public health, VHA, and Kaiser Permanente). One of the educational products from this collaboration was launching a new graduate course in the multi-disciplinary clinical sciences graduate program. The training materials and workbook resources were published online and disseminated via the NIH CTSA network to increase spread in hope that they might serve as a resource for other local D&I training workshops. To achieve reach among those who did not attend the workshop, an interactive e-book format for the workbook was also launched Fall 2014.
